# PDE7 as a Precision Target: Bridging Disease Modulation and Potential PET Imaging for Translational Medicine

**DOI:** 10.1021/acsmedchemlett.5c00160

**Published:** 2025-04-15

**Authors:** Taoqian Zhao, Steven H. Liang

**Affiliations:** Department of Radiology and Imaging Sciences, 1371 Emory University, Atlanta, Georgia 30322, United States

**Keywords:** Phosphodiesterase 7 (PDE7), cAMP-PKA signaling, Pyrimidinone, PET imaging

## Abstract

Phosphodiesterase 7 (PDE7) regulates cAMP-PKA signaling and plays a crucial role in immune function, neuroprotection, and inflammation. Dysregulated PDE7 activity is linked to neurodegenerative, autoimmune, and metabolic disorders, making it a promising therapeutic target. Recent advancements in PDE7 inhibitors, particularly pyrimidinone-based compounds, have shown high selectivity and potent biological effects. Beyond therapeutics, radiolabeled PDE7 inhibitors offer potential for PET imaging, enabling noninvasive disease monitoring and treatment assessment.

## Graphical abstract

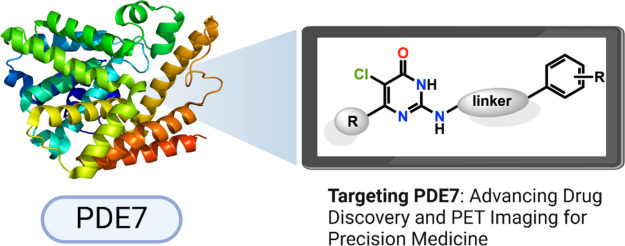

Cyclic nucleotide signaling is a fundamental regulatory mechanism that governs various physiological processes by orchestrating cellular responses through complex molecular networks.^1^ Among these, the cAMP-PKA (cyclic adenosine monophosphate–protein kinase A) pathway is a key regulatory node that modulates cellular responses to external stimuli. This pathway is initiated when G protein-coupled receptors (GPCRs) activate adenylyl cyclase, an enzyme that catalyzes the conversion of ATP into cAMP. The resulting increase in cAMP levels promotes the activation of PKA, which exists in an inactive state as a complex of regulatory and catalytic subunits. Binding cAMP to the regulatory subunits induces the release of the catalytic subunits, transitioning PKA into its active form, which phosphorylates downstream targets to regulate gene expression, metabolism, and immune responses (Figure 1A).^2,3^ To maintain cellular homeostasis, phosphodiesterases (PDEs) hydrolyze cAMP, effectively terminating PKA signaling.^4^

**Figure 1:**
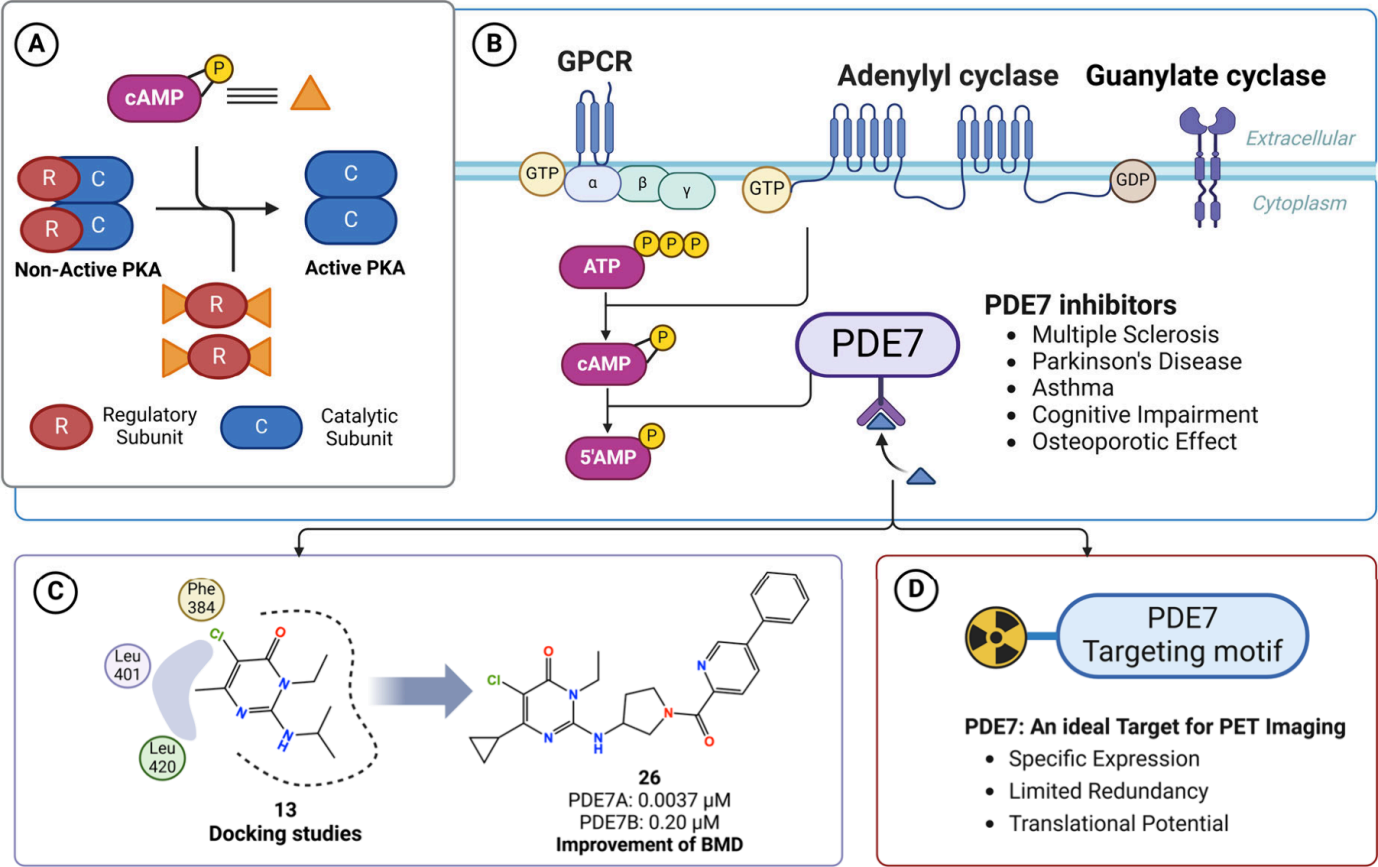
PDE7 in cAMP signaling, therapeutic targeting, and PET imaging potential. (A) PKA activation via cAMP binding. (B) PDE7 regulates cAMP-PKA signaling, influencing immune and neurological functions. (C) Structural optimization for improved bone mineral density (BMD). (D) PDE7 as a promising PET imaging target due to specific expression and translational potential.

Among the 11 phosphodiesterase (PDE) families, PDE7 is a cAMP-specific enzyme that plays a pivotal role in immune regulation, neuroprotection, and inflammatory responses. By selectively degrading cAMP, PDE7 serves as a key modulator of the cAMP-PKA signaling pathway, counterbalancing adenylyl cyclase activity and preventing excessive downstream activation (Figure 1B). Dysregulation of PDE7 has been implicated in neurodegenerative diseases, autoimmune disorders, chronic inflammation, and metabolic dysfunctions, as elevated PDE7 activity reduces intracellular cAMP levels, impairing protective mechanisms that depend on sustained PKA activation.^5,6^ Consequently, PDE7 inhibitors have emerged as promising therapeutic agents for conditions such as multiple sclerosis, Alzheimer’s disease, Parkinson’s disease, asthma, cognitive impairment, and osteoporosis, aiming to restore cAMP homeostasis and regulate immune responses. PDE7 exists in two isoforms, PDE7A and PDE7B, with distinct expression patterns and functional roles. PDE7A is predominantly expressed in the lungs, hematopoietic cells, and placenta, suggesting a role in immune function and respiratory health. In contrast, PDE7B is widely distributed across various tissues, including the pancreas, brain, thyroid, and skeletal muscle, indicating broader physiological functions beyond immune regulation.^7^ The differential expression of these isoforms underscores the tissue-specific regulation of cAMP signaling and highlights the potential for selective PDE7 inhibition as a targeted therapeutic strategy.

Over the past decades, significant progress in medicinal chemistry has led to the development of highly potent PDE4 inhibitors, such as Cilomilast and Roflumilast, which have undergone clinical trials for asthma and chronic obstructive pulmonary disease (COPD). However, despite their therapeutic potential, only a few PDE4 inhibitors have received market approval, largely due to their narrow therapeutic window and dose-limiting adverse effects such as emesis, which restrict their clinical utility.^8^ As an alternative approach, targeting another cAMP-specific phosphodiesterase, PDE7, has gained attention, given its expression in immune and pro-inflammatory cells and its involvement in inflammatory and neurodegenerative diseases.

Recent advancements in PDE7 research indicate that PDE7A and PDE7B share highly similar catalytic domains and exhibit comparable high affinity for cAMP, making the development of selective PDE7 inhibitors an achievable goal. Structural and computational studies have provided valuable insights into PDE7 inhibition, guiding the rational design of highly selective and potent small-molecule inhibitors.^9,10^

Given the structural similarities with cAMP and cGMP, nucleobase-mimicking scaffolds such as pyrimidinones and fused pyrimidinones have emerged as promising core structures for PDE inhibitors. Leveraging three-dimensional structural data of PDE7A and PDE7B, Kentaro et al. identified a novel PDE7A inhibitor with high selectivity over closely related isoforms (Figure 1C).^11^ During the optimization process, compound 13 demonstrated the highest potent PDE7A inhibitory potency among a series of 10 analogs. Docking studies revealed that its methyl substituent in compound 13 did not fully occupy the binding pocket within the PDE7A active site (Figure 2). To enhance potency and selectivity, the introduction of a bulkier cyclopropyl group led to a 20-fold increase in PDE7A inhibitory potency while preserving its activity against PDE7B. Further modifications explored the distal group by incorporating a phenyl ring connected via a conformationally constrained linker, ultimately yielding the most potent and selective PDE7A inhibitor with an IC_50_ of 0.0037 μM and a 54-fold selectivity over PDE7B (IC_50_ = 0.20 μM).

**Figure 2:**
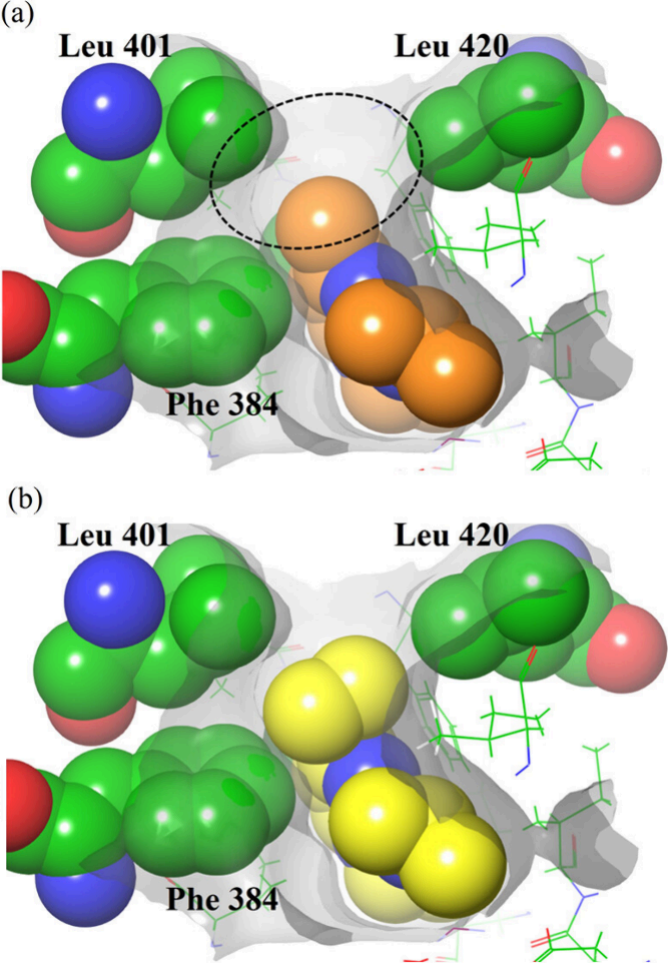
Docking conformations of (a) compound 13 and (b) compound 14, where the methyl group is replaced with a cyclopropyl group for improved binding. The dashed circle highlights an unoccupied space near the methyl group in compound 13, indicating a potential site for structural optimization to enhance PDE7A selectivity and potency. Reproduced with permission from ref 11. Copyright 2024 American Chemical Society.

Beyond its enzymatic potency, the optimized PDE7 inhibitor demonstrated significant biological effects, particularly in bone metabolism. Oral administration (30 mg/kg) significantly improved bone mineral density (BMD), achieving efficacy comparable to that of 5 μg/kg hPTH(1–34), a clinically relevant dose in humans (Figure 3). These findings highlight the therapeutic potential of PDE7 inhibitors in not only inflammatory and neurological disorders but also bone-related conditions.

**Figure 3:**
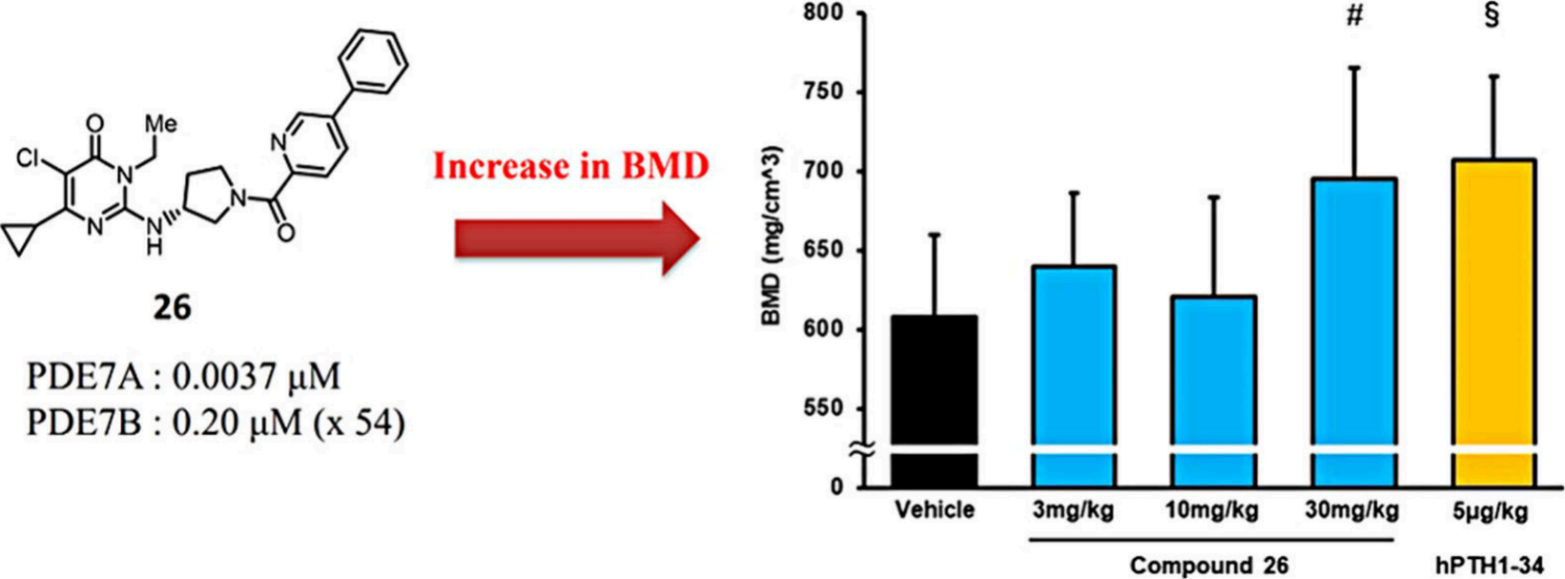
Evaluation of PDE7A-selective inhibitor **26** on Bone Mineral Density (BMD). The chemical structure of compound **26** is shown, along with its high selectivity for PDE7A (IC_50_ = 0.0037 μM) over PDE7B (IC_50_ = 0.20 μM, 54-fold selectivity). The bar graph illustrates the dose-dependent increase in BMD following administration of compound 26 (3, 10, and 30 mg/kg) compared to the vehicle control. Reproduced with permission from ref 11. Copyright 2024 American Chemical Society.

## Outlook

Selective PDE7 inhibitors have emerged as promising therapeutic agents due to their enhanced isoform specificity, optimized pharmacokinetic profiles, and improved therapeutic efficacy. Precise modulation of PDE7A and PDE7B strengthens their role in cAMP signaling regulation, offering promising applications in neurodegenerative, autoimmune, and inflammatory diseases, as well as reinforcing their role in precision medicine and targeted therapy development.

Positron emission tomography (PET) is a powerful molecular imaging technique that enables in vivo visualization and quantification of specific biological targets.^12,13^ Beyond their therapeutic potential, PDE7 inhibitors also show promise as PET imaging agents.^14,15^ Differential PDE7 expression in immune cells, the central nervous system, and inflammatory sites allows for noninvasive disease monitoring. Several radiolabeled PDE7 ligands, including [^11^C]­MTP-38,^16^ [^18^F]­MICA-003,^17^ [^11^C]­P7-2104,^18^ and [^18^F]­P7-2302,^19^ have been developed. Although these ligands exhibit limitations, such as insufficient specific bindings and/or poor brain permeability, they represent initial efforts to enable real-time imaging of PDE7 in the brain. The successful development of an optimized PDE7 PET ligand would provide valuable insights into disease progression, treatment response, and patient stratification in conditions such as Alzheimer’s disease, Parkinson’s disease, multiple sclerosis, and chronic inflammation.

Structural insights gained from PDE7 inhibitor optimization can further guide the design of PET tracers with high binding affinity, metabolic stability, and blood–brain barrier permeability. Radiolabeled PDE7 inhibitors could be particularly useful in neurodegenerative diseases, where visualizing PDE7 expression in affected brain regions may aid in early diagnosis and therapeutic monitoring.

In conclusion, the development of selective PDE7 inhibitors represents a promising therapeutic strategy for treating inflammatory, neurological, and metabolic disorders. Advances in structure-based drug design have led to the identification of potent and selective PDE7A inhibitors, with notable biological effects, including enhanced bone mineral density. Moving forward, radiolabeled PDE7 inhibitors hold significant potential as PET imaging agents, providing a powerful tool for disease monitoring, drug evaluation, and precision medicine. Further research into pharmacokinetics and biodistribution of PDE7-targeted PET tracers will be essential for translating these findings into clinical applications.

## Abbreviations

PDE: phosphodiesterase
cAMP: cyclic adenosine monophosphate
PKA: protein kinase A
GPCR: G protein-coupled receptors
ATP: Adenosine triphosphate
BMD: bone mineral density
PET: positron emission tomography.
